# Supplementary material for: Are carbon emissions trading and green financial instruments synergistic? -Comprehensive quantitative research based on content analysis
Source: PLoS One. 2024 Mar 7;19(3):e0298601. doi: 10.1371/journal.pone.0298601 (PMC10919601; doi:10.1371/journal.pone.0298601)
Supplement: S1 Data — (ZIP) [file pone.0298601.s001.zip › Data/地级市Z_F工作报告.zip!/─┌├╔╣┼/╢⌡╢√╢α╦╣╩╨/└┤╫╘╣1⁄2╓┌║┼í╛┬φ┐╦╩2╛▌═°í┐_2019.docx]

各位代表：

现在，我代表旗人民政府向大会报告工作，请予审议，并请政协委员和列席会议的同志们提出意见。

一、2018年工作回顾

过去的一年，是新一届政府履职的开局之年，也是攻坚克难、砥砺奋进、取得丰硕成果的一年。一年来，在市委、市政府和旗委的坚强领导下，在旗人大和政协的监督支持下，旗人民政府团结带领全旗各族人民，围绕建设富饶、友善、美丽达拉特，统筹推进改革发展稳定各项工作，较好地完成了十七届人大二次会议确定的目标任务，经济社会保持平稳健康发展态势。预计完成地区生产总值366.7亿元，同比增长6%；一般公共预算收入18.8亿元，增长44.6%；固定资产投资101.3亿元，增长6.2%；社会消费品零售总额67.4亿元，增长7%，综合实力位列中国西部县域经济百强第28位和全国工业百强县市第78位。

一年来，我们坚持转方式、调结构、促转型，高质量发展迈出新步伐。现代农牧业提质增效。突出龙头企业带动，推出代耕代种代养等新型经营模式，完成节水农业项目3.3万亩，新增市级以上龙头企业5家，发展订单农业55万亩，农企利益联结率达65%。新认证有机农产品10个，生产绿色有机农产品16万吨，荣膺自治区农畜产品质量安全监管示范旗。农牧业再获丰收、结构稳中调优，粮食产量连续14年突破十亿斤，粮经饲比例优化为56∶21∶23；鹌鹑、南美白对虾等特色养殖效益明显，鲜奶、肉类产量达到18.2万吨和4.5万吨。工业经济持续向好。规模以上工业增加值、工业用电量分别增长10%和43.5%，全年火电机组利用小时数4700小时、发电196.6亿度，生产原煤突破5000万吨、甲醇182万吨、PVC50.2万吨、氧化铝38万吨。非煤产业投资占到工业总投资的90.7%，蒙泰18万吨铝板带等项目建成投产，荣信化工二期、新奥石墨烯等项目进展顺利，全国集中度最高、规模最大的沙漠光伏发电应用领跑基地一期500兆瓦项目，实现一次性全容量并网发电。开发区实现工业总产值170亿元，增长37%。现代服务业亮点纷呈。开展“旅游品质提升年”行动，文旅创投基地、林原村乡村游服务中心启动运营，响沙湾成为全区优秀民营企业和旅游厕所建设先进典型，成功举办大型主题活动16场次，旅游人数、收入分别增长16.4%和12.9%。开发区综合物流园区规划全面启动，荣通等煤炭物流园区发运煤炭6200万吨；公路、铁路货运量分别增长21%和75%。建成苏木镇和村级电商服务站55个、行政村物流投送点132个，电商物流成本下降40%，全年实现电商交易额1.6亿元，增长7%。

一年来，我们坚持出重拳、强举措、攻难点，“三大攻坚战”开局良好。财政金融风险有序化解。多措并举化解政府债务30.4亿元，完成年度化债任务的144.7%，其中化解“十个全覆盖”工程债务16.5亿元。处置银行不良贷款9.5亿元，审结非法集资案件7起、涉案金额3.1亿元，有效维护了金融稳定。脱贫攻坚成效显著。223户475名建档立卡贫困人口脱贫；各级财政投入扶贫资金1.1亿元，全年发放产业扶持资金1808万元、金融扶贫贷款4267.5万元，筹集社会扶贫善款659万元；建档立卡贫困户与企业实现100%利益联结；全面落实健康扶贫“六重”保障政策，贫困人口医药费报销比例达91.3%。环境质量明显改善。中央环保督察反馈问题按时限完成整改、“回头看”转办举报问题全部办结。亿利、新能煤场全封闭和高盐水结晶项目全面完工，取缔城区燃煤小锅炉163台，清理城区废品收购站点104处、养殖场25个，完成平房区1134户居民集中供热改造；出台环境综合治理标准，开展了煤矿、煤场、非煤矿山、交通沿线环境集中整治，全旗空气质量优良率由76.5%提高到79.6%。工业固废处理、排污口治理扎实推进，农业面源污染得到有效控制。科学划定了生态红线范围。完成林业生态工程13.3万亩、水保综合治理29万亩、矿区复垦绿化1.1万亩。

一年来，我们坚持抓改革、求创新、促开放，地区发展动能有效释放。深化供给侧结构性改革，降低企业各类成本2.4亿元，消化存量商品房21.1万平方米。“放管服”改革深入实施，取消行政审批事项86项，网上预审事项达370项，办理时限普遍压缩30%以上。国家增量配电业务改革试点实现实质化运营，土地草牧场确权颁证基本完成，内置金融、校园足球等一批改革亮点得到上级肯定，国有林场改革、供销社综合改革等重点改革取得新成效。设立了科技创新奖励资金，新增国家高新技术企业2家，转化运用科技成果15项，建成国家级星创天地2家，入选国家“万人计划”科技创业领军人才1人。营商环境全面优化，新增各类市场主体4022户，新签约项目25项。实施亿元以上重点项目28项，完成投资91亿元。积极开拓国外市场，完成出口额4500万美元。

一年来，我们坚持抓统筹、补短板、强基础，城乡发展面貌持续改观。完成城市空间总体策划编制和5条街道街景设计，调整划定了城市棚改区域和城市核心区范围。着力补齐市政设施短板，解决了一批长期影响城区道路环通、景观提升的“老大难”问题，新建改造南园街、树林召大街、西园路等市政道路9.3公里、排水管网11.7公里，实施街巷硬化6.5万平方米，新增更换路灯608套，改造节能路灯4698盏；新建水冲式和移动环保式公厕21座，城区机扫率由40%提高至60.5%。实施城区景观节点9处，新建城市绿道5.2公里，改造提升绿化景观40万平方米。化肥厂和兴盛园片区棚改项目完成房屋征收129户，人和二期安置小区安置棚改回迁户115户。乡村振兴开局良好，制定出台了实施意见和行动方案；132个嘎查村建立项目库，村集体经济收入达到861万元，树林召镇东海心村获评全国“一村一品”示范村；建成低温裂解生活垃圾处理试点1处、村级污水处理试点2个，完成户改厕1700户，成为全区农村环境综合整治试点旗。基础设施不断完善，黄河二期防洪工程全面完工；G65改扩建工程进展顺利，沿黄一级公路按时实现封闭，荣获“四好农村路”全国示范县称号；电信普遍服务试点、中心村农网改造项目全面完工，城网改造工程完成投资1.5亿元。

一年来，我们坚持惠民生、保稳定、促和谐，群众幸福指数稳步提升。完成民生及社会事业投入43.9亿元，占财政支出的87%。城乡居民收入分别增长7.2%和9%。第八小学综合楼等3个项目投入使用，城区中小学班容量持续下降；新招聘教师270名，继续实行教师交流轮岗等制度；高考本科上线率提高到72.5%，义务教育综合考评位居全市前列，被教育部列为校园足球“满天星训练营”和全国校园足球优秀试点县。新组建医联体、专科联盟18个，分级诊疗体系基本建立，城乡居民家庭医生签约率达49.8%，完成8400名适龄妇女“两癌”免费筛查。新创作“五个一”精品文艺作品40件，组织大型群众文化活动33场次，旗图书馆荣膺国家县级一级图书馆，乌兰牧骑获评自治区一类乌兰牧骑。新增城镇就业4205人，城镇登记失业率控制在3.1%以内。企业退休人员和城乡居民养老金每人每月提高141元和118元，城乡低保标准每人每月提高45元和37元，农村特困人员集中和分散供养标准每人每年提高1000元和588元。发放城乡低保、抗灾救灾等民生类资金7.1亿元。设立退役军人基层服务管理站和服务办，发放各类补贴2537万元。创新社会治理，“3+X”基层网格化治理试点成效显著。严格落实信访工作责任制，信访批次和人次实现“双下降”。开展常态化安全生产大检查，安全生产形势总体稳定。落实“四个最严”要求，保障了群众“舌尖上的安全”。防凌防汛取得全面胜利，成功应对黄河长时间、大流量运行。动物疫病防控全面加强，全旗未发生非洲猪瘟疫情。建成标准化防雹作业点23处。深入推进扫黑除恶专项斗争，严厉打击违法犯罪活动，社会治安形势持续向好。

一年来，我们坚持转作风、提效能、强落实，政府自身建设全面加强。严守政治纪律规矩，加强政府系统党风廉政建设，扎实推进“两学一做”学习教育常态化制度化，全面开展“干部队伍建设年”活动，政府行政能力持续提升。健全政府党组工作规则，严格执行“三重一大”决策和政府法律顾问制度。自觉接受人大法律监督和政协民主监督，115件人大代表建议和151件政协委员提案全部答复。强化督促检查和跟踪问效，全力抓好巡视巡察问题整改。严格执行中央八项规定和国务院“约法三章”，持之以恒纠正“四风”，政府会议和文件数量持续下降。坚持以“零容忍”态度惩治贪腐，集中整治政府系统“雁过拔毛”式腐败问题，共立案38件，给予党纪政务处分12人。同时，精神文明、民主法治、人民武装、国防动员、民族宗教、新闻广电、外事侨务、史志档案、气象地震、妇女儿童等工作均取得新成绩。

各位代表，刚刚过去的2018年，是大事多、要事多、喜事也多的一年。我们通过全国人大常委会大气污染防治执法检查，栗战书委员长对我们的工作给予充分肯定，给全旗人民带来极大鼓舞和激励，凝聚起了干事创业的强大正能量；我们接受中央媒体代表团集中采访，向全国展示了库布其沙漠治理模式，治理经验成为全国生态文明建设重大典型，极大提振了全旗人民坚持绿色发展的信心和决心；我们以高度的政治自觉，全力抓重点、攻难点、创亮点，聚焦聚力打好三大攻坚战，经济社会发展实现稳中提质，朝着实现全面建成小康社会目标迈出了坚实步伐。这些成绩的取得，是旗委正确领导的结果，是旗人大、政协鼎力支持的结果，也是全旗各族人民合力攻坚、拼搏奋斗的结果。在此，我代表旗人民政府，向全旗各族干部群众，向所有关心支持达拉特发展的社会各界人士，表示衷心的感谢并致以崇高的敬意！

在看到成绩的同时，我们也清醒地认识到，前进的道路中仍有不少困难和挑战。主要表现在：经济发展质量不高、动能不足，推动高质量发展任重道远；农牧业种养加销全产业链尚未完全建立，工业“原字头”企业多、产品附加值低、战略性新兴产业比重小，旅游、物流产业潜力和效益尚未充分释放，产业“四多四少”问题依然突出；项目储备不足，服务项目不到位，推动新旧动能转换的大项目、好项目不多；城市基础设施存在短板，城市功能和面貌与旅游中心城市定位不相匹配；农村厕所、垃圾污水处理等设施不完善，农牧民思想教育亟待加强，推进乡村振兴任务艰巨；教育、卫生、文化等公共服务相对滞后，财政收支矛盾突出，生态建设、社会治理任务依然繁重；部分干部不善为、不能为、不作为现象还不同程度存在。对这些问题，我们将精准施策、持续用力，认真加以解决。

二、2019年工作安排

今年是新中国成立70周年，也是全面建成小康社会关键之年，做好全年工作至关重要。我们将精准聚焦旗委把2019年确定为“重点工作突破年”的主攻方向，始终保持勇立潮头的闯劲、攻坚克难的韧劲、为民造福的干劲，全力以赴，狠抓落实，朝着推动高质量发展、决胜全面小康目标奋勇前进。今年政府工作的总体思路是：以习近平新时代中国特色社会主义思想为指导，深入贯彻党的十九大和十九届二中、三中全会精神，全面落实中央、自治区、市各项决策部署，统筹推进“五位一体”总体布局，协调推进“四个全面”战略布局，坚持稳中求进工作总基调，全面践行新发展理念，聚焦聚力高质量发展，坚持以供给侧结构性改革为主线，做大经济总量，提高发展质量，加快建设现代化经济体系，继续打好三大攻坚战，统筹做好稳增长、促改革、调结构、惠民生、防风险工作，推动经济发展质量变革、效率变革、动力变革，加快建设富饶、友善、美丽达拉特，全力保持经济持续健康发展和社会大局和谐稳定，为全面建成小康社会收官打下决定性基础。

主要预期目标是：地区生产总值增长6%左右，一般公共预算收入增长3%左右，固定资产投资增长10%以上，社会消费品零售总额增长7%左右，城乡常住居民人均可支配收入分别增长7%和8%左右。

围绕上述目标，重点抓好七个方面的工作：

（一）坚定不移打好三大攻坚战，在夯实全面建成小康社会基础上迈出新步伐

主动防范化解重大风险。着力抓好增收节支工作。精心培育新的税源，从增强地方财力入手构筑项目、发展产业，以税源结构的调整和建设形成新的经济增长点。有效处置盘活存量用地、边角煤等资源资产，积极筹措化债资金。统筹使用好财政资金，按5%比例压减一般性支出，集中有限财力保工资、保运转、保民生。着力强化政府债务管理。综合运用预算安排、资产处置、清欠税费等措施，完成7.67亿元年度化债任务。正确处理发展与化债的关系，坚持开前门、堵后门，年内向上争取转移性支付和债券资金5亿元以上，严格规范政府举债融资、基金运作、政府购买服务行为，坚决杜绝新增隐性债务。着力防控金融领域风险。强化地方法人金融机构监管，全面规范经营行为。健全金融风险监测预警和应急处置机制，支持金融机构依法处置不良贷款，加快非法集资涉案资产处置。严厉打击非法集资、金融诈骗等违法犯罪活动，优化地区金融生态。

精准发力推进脱贫攻坚。围绕“两不愁、三保障”目标要求，统筹抓好贫困户、巩固户、边缘户帮扶工作，确保75户175名贫困人口脱贫，贫困发生率降至0.1%以下。着力推进“开发式”扶贫。突出抓好产业扶贫，继续推广订单包销、定额分红等扶贫模式，创新推行“抓村带户”产业扶贫模式，逐村逐片确定主导产业、建立新型经营机制，推动扶贫产业持续发展、长效惠民。着力推进“保障式”扶贫。落实健康扶贫“六重”保障和“三个一批”政策，贫困人口医药费报销比例稳定在90%以上；采用引入商业保险等手段，建立非贫困人口大病救助保障机制，降低因病致贫发生率。全力帮助贫困户在公益性岗位就业，加强无劳动能力贫困人口兜底保障。着力推进“全民式”扶贫。深入开展定点帮扶工作，推进“百企帮百村”行动，发挥社会扶贫平台作用，组织开展各类活动，引导全社会力量参与扶贫。着力推进“励志式”扶贫。坚持志智双扶，做好思想教育、技能培训等工作，落实“奖帮歇帮”制度，激发贫困户内生动力。坚持以最有力的举措，抓好扶贫领域巡视反馈问题整改落实。

下大力气进行污染防治。按照坚守阵地、巩固成果要求，推动自治区生态大检查和中央环保督察及“回头看”反馈问题整改，落实好年度污染防治任务。坚决打赢蓝天保卫战。推进工业园区、矿区、交通沿线环境整治常态化，启动大车物流园区建设，加强城郊和绕城路大型货车扬尘污染治理；强化散煤燃烧和燃煤锅炉整治，实施城市平房区供热改造工程，启动苏木镇煤改电供热改造；推进工业污染源达标排放，实施重点企业粉状物料堆场全封闭工程，确保空气质量优良率达到80%以上。着力打好碧水保卫战。加强饮用水水源地保护，加快树林召等3个地下水超采区综合治理工程建设；推进荣信化工高盐水结晶项目，提高中水回用率；落实旗乡两级河长责任，全力保障水环境安全。扎实推进净土保卫战。加强土壤污染管控和修复，完成土壤污染状况详查，建立农用地土壤环境质量分类清单；推进“三废”循环再利用，确保工业固废和危险废物安全处置。加强环保网格化管理，强化一体化综合执法，严厉查处环境违法行为。

（二）坚定不移抓项目扩投资优环境，在助推经济高质量发展上迈出新步伐

狠抓重点项目建设。把项目建设作为推动经济高质量发展的“牛鼻子”，聚焦改变产业“四多四少”状况，实施500万元以上重大项目122项，计划完成投资110亿元以上，年内确保盛德源二甲醚、东方希望生猪养殖、马场壕铁路集运站等71个项目建成投用，形成支撑经济高质量发展的硬实力。继续实行“五个一”项目推进机制，严格执行项目审批领办、带办和限时办结制，倒排工期、挂图作战，确保项目早获批、早开工、早投产。准确把握国家产业政策和投资导向，认真做好项目策划和储备，积极争取上级政策和资金支持，有效撬动社会资本参与投资。

加大招商引资力度。突出产业链招商，立足煤化工、煤电铝等产业基础，引进增链、补链、强链项目，打造产业配套、产品对接、优势互补的产业集群。突出以商招商，引导中小企业围绕主导产业搞延伸，围绕重点项目搞协作，围绕骨干企业搞配套，形成优势互补新格局。突出全民招商，出台招商引资奖励办法，实行招商引资项目管理制和责任人负责制，营造“人人抓招商、个个引项目”的良好氛围。年内引进亿元以上重大项目10项以上、国内区外资金70亿元以上。

全力推进园区振兴。加快开发区调区扩区进度，完成园区总规和产业规划修编，加快建设园中园、特色园，促进产业规模化、循环化发展，把园区建成高端要素集聚区和高质量发展引领区。保障国家增量配电业务改革试点稳定运营，形成电价洼地效应，增强园区产业吸附力。积极清理园区“僵尸企业”，腾出更多的要素指标和发展空间保障优质项目。完善园区基础设施，新建园区道路4.7公里、供电线路10公里，加快达电等企业余热循环利用，提升园区服务承载能力，年内开发区工业总产值突破200亿元。

持续优化营商环境。树立“服务至上”理念，巩固扩大“营商环境建设年”活动成果，打造亲商、安商、敬商的全市一流营商环境，助推经济高质量发展。深化“放管服”改革，推进“互联网+政务服务”，加快“一网、一门、一次”政务服务改革，确保政务服务事项网上可办率在70%以上，“最多跑一次”事项达到100项以上。优化服务企业机制，实行部门服务承诺制，建立服务项目“绿色通道”，为项目建设提供全程跟踪服务。大力弘扬企业家精神，依法保护企业家人身和财产安全，构建亲清政商关系。

大力支持民营经济。认真落实中央、自治区和市支持民营经济发展的政策措施，做大做实做优民营经济。全面落实国家减税降费政策，规范清理各类涉企收费，降低企业税费成本。设立产业发展引导基金，鼓励金融资金和社会资本扶持实体经济发展。积极帮助民营企业解决历史遗留问题，有序化解政府部门和旗属国有企业拖欠民营企业款项问题。落实“助保贷”等金融扶持政策，有效解决民营企业融资难融资贵问题。

（三）坚定不移推动产业转型升级，在培育新产业新动能新增长极上迈出新步伐

加快发展现代能源经济。推动能源化工产业精细化发展。坚持以煤为基、多元发展，加快煤化工产业链条延伸，确保荣信化工乙二醇等项目建成投产，推进信耐醋酸项目建设，争取君正烯烃、北斗航天甲醇制混合芳烃等项目落地，年内新增现代煤化工产能65万吨以上。推动清洁能源产业绿色化发展。加快沙漠清洁能源与沙漠农林、沙漠特色旅游等产业融合发展，确保光伏基地一期500兆瓦光伏发电项目稳定运行，启动二期500兆瓦项目建设，打造国内最大的集中式光伏发电和清洁能源经济示范基地；围绕“光伏+旅游”实施好光能环旅游项目，通过“林光互补、农光互补”栽植平铺式沙柳束1.2万亩，种植矮化密植红枣等经济作物8500亩，加快建设“金沙、蓝海、绿洲”沙漠经济先导区。推动煤电铝产业一体化发展。运用新技术、新业态、新模式，加快煤炭产业改造升级，推动企业兼并重组和上下游融合发展，促进煤炭资源就地转化，提高煤炭清洁、高效、集约利用水平，加快电力产业绿色化发展，力争原煤洗选率稳定在92%以上、煤炭就地转化率达到50%以上、火电机组年利用小时数在5000小时以上。加快铝产业发展，规划建设铝产业园，确保鑫旺氧化铝、蒙泰铝板带、新长江高纯铝项目稳定生产，争取中铝电解铝项目落地开工，不断延长产业链，提升产品附加值。

做大做强战略性新兴产业。建立推动新兴产业高质量发展保障体系，注重核心技术研发和应用，加快发展新材料产业，开工建设恒星有机硅聚合物项目，推进索能碳化硅等项目建设，推动新奥石墨烯产品链条化、终端化，培育壮大经济发展新动能。大力发展节能环保产业，推动共同低碳可燃驰放气循环再利用等项目建设，确保建能兴辉绿色发泡陶瓷及复合墙体材料生产线建成投产，尽快形成新的经济增长点。

推动服务业提档升级。壮大文化旅游业。实施全域旅游发展战略，推进旅游资源“点线面”有机统一，形成“吃住行、游购娱”综合发展的“旅游+”和“+旅游”，打造国内外沙漠度假、户外运动、乡村旅游知名旅游目的地。推动景区景点品质提升，完成响沙湾休闲度假环境提升和响沙湾港改扩建工程，实施库布其国家沙漠步道户外运动公园、王爱召民族文化旅游区等重点项目，打造高品质乡村旅游示范村3个、乡村旅游示范户5家。编制全域旅游公共服务体系规划，力争启动建设文旅综合服务中心，打通便捷的旅游交通网络，新建改造一批高标准旅游厕所，实施“手游达拉特”智慧旅游工程，办好黄河几字湾开河节和冬捕节、戈壁天堂文化艺术节、勇士文化节等品牌节庆活动，加强线上线下宣传营销，推出研学旅行、沙漠探险等旅游新产品，打造一批沙漠体验、生态度假等精品旅游线路，增强游客参与度和可停留性，确保旅游人数和收入实现两位数增长。构建现代物流体系。编制现代物流产业规划，出台促进物流业高质量发展的政策措施，加强物流业管理整合。开工建设开发区综合物流园区，搭建煤炭、化工、建材等大宗工业产品交易平台，打造国家物流枢纽承载基地和西部地区大宗商品物流交易中心。提升荣通、联创等煤炭物流园区功能，推进青达门矿区物流小镇、点石沟铁路集运站建设，加快建设“鄂尔多斯煤超市”。大力发展电子商务。巩固全国电商进农村综合示范县创建成果，优化电商基地和地方特色体验馆功能，新建嘎查村、社区电商服务站各10个，培育农村电商示范村2个，通过线上线下销售让地方优质产品打开市场、卖出好价。继续推进“万户企业登云计划”，提高工业企业电商普及率，加快发展工业品电商和跨境电商。年内实现电商销售额2亿元，增长25%。

（四）坚定不移落实乡村振兴战略，在加快农业农村繁荣发展上迈出新步伐

聚焦产业振兴，推动农牧业高质量发展。深化农牧业供给侧结构性改革，实施农牧业高质量发展十大行动，深入推进一二三产业融合发展，加快建设“北纬40度、黄河几字湾”健康安全农畜产品生产加工输出基地。夯实农牧业基础。实施昭君镇灌域排水等高标准农田项目10万亩，首期补充耕地库项目2万亩，改造沿河未利用盐碱地1万亩以上；新建农机示范园区3个，农业综合机械化水平提高到94%；完善农牧业科技推广服务体系，主要农作物良种使用率达到100%，新技术普及率达到90%以上；新建基层供销社3个，规划建设羊绒特色小镇；严格落实耕地保护制度，推进“大棚房”问题清理整治工作。培育壮大新型经营主体。培育市级以上龙头企业3家，发展示范性合作社50个、家庭农牧场100家，培养新型职业农牧民200人。引导土地草牧场向龙头企业适度集中，落实订单农业60万亩，适度规模经营70万亩以上，农企利益联结率提高到68%以上。推动种养加销一体化。围绕玉米、乳肉、果蔬等主导产业，加快组建一批产业化联合体，促进种养加销全产业链发展。优化“粮经饲”结构，推广种植鲜食玉米、粮饲兼用等多用途玉米，打造优质绿色瓜果蔬菜规模化主产区，饲草料种植稳定在50万亩以上。围绕建设养殖大旗，加快落实奶牛肉牛养殖业发展规划，建设奶牛标准化养殖国家级示范牧场1家、自治区级示范牧场2家，新建标准化奶牛肉牛养殖场3个、生猪养殖园区2个；开展陆基集装箱养鱼等新技术养殖试点。提升风水梁等现有农畜产品园区功能，力争启动呼宝鳄鳄鱼产业园和农畜产品生产加工物流基地建设，加快构建“一核五区”绿色物流体系。实施“互联网+农牧业”行动，建立农业大数据平台，健全农畜产品销售网络体系。落实品牌强农战略。坚持质量兴农、绿色兴农，年内农作物标准化种植达到100万亩以上，“三品”认证农产品总量达到38万吨。开展农牧业产地环境净化行动，完善农畜产品生产加工标准和质量安全追溯体系，推动国家农产品质量安全监管示范县创建，增加优质绿色农畜产品供给。

聚焦美丽宜居，加快农村牧区现代化。围绕推动乡村振兴“六大行动”，完善旗乡两级乡村振兴实施方案，完成苏木镇总规、示范村建设规划编制，组织实施16个乡村振兴示范村建设。启动黄河湿地农业田园综合体建设，推动特色种养、休闲旅游、创意农业等新业态一体化发展。发展壮大村集体经济，年内收入突破1000万元。突出抓好农村垃圾污水处理、厕所革命、村容村貌整治提升，健全垃圾收集、储运、处理长效机制，新建新型生活垃圾处理站20处、污水处理项目2个，完成户改厕2.2万户，着力补齐影响农牧民生活品质的短板。严控农作物秸秆进村入户，推动秸秆“五化”利用，确保秸秆综合利用率达到85%以上。出台农业面源污染治理实施细则，深入开展“四控行动”；利用先进生物技术推进畜禽养殖废弃物资源化利用，争创粪污资源化利用整县推进聊渴痉镀臁Ｊ凳傲焱费恪焙陀判闳瞬呕叵绱匆导苹嘤慌缤寥瞬藕椭赂淮啡恕Ｈ嫱菩小?+X”基层网格化治理模式，完善村规民约和“红黑榜”道德诚信体系，调动镇村社三级网格员积极性，加快基层服务管理标准化、精细化。实施乡村优秀传统文化传承、乡风文明培育、农牧民思想教育“三大工程”，深化干净人家、敬老孝星等评比活动，让崇尚感恩、尊老爱幼、向善向美成为社会风尚。

聚焦生态振兴，加大生态保护建设力度。坚持绿色富旗、绿色富民，统筹山水林田湖草系统治理，大力弘扬库布其精神，实施京津风沙源治理二期、十大孔兑沙棘生态减沙等重点生态工程，完成林业生态建设11.1万亩，新增水保综合治理25万亩。落实农村重点区域、交通沿线绿化管护主体，加大村庄、城郊等地区造林绿化力度，启动“四大绿色走廊”建设。围绕绿色矿山建设，完成20处露天煤矿、3000亩的复垦绿化。坚定不移执行禁牧禁垦政策，严厉打击违法开荒和破坏林地草原行为，坚决守住生态底线。

（五）坚定不移推进新型城镇化，在推动城乡区域协调发展上迈出新步伐

开展城市品质提升行动。按照“收缩限扩”总体思路，从微观领域破题、群众身边事入手，着力优化城市布局、完善城市功能、提升城市品位，加快建设内蒙古旅游中心城市。着力优化城市规划体系。完成新一轮城市总规修编和控规审批，编制城市空间发展规划、近期建设规划和棚户区改造策划，完成迎宾街等7条主街道街景设计。着力完善市政基础设施。下大力气环通城市主干道，持续优化城区路网，确保南园街东段、建设路和召西路南段等市政道路全线通车，同步推进新华路等主街道提升改造，新建改造市政道路15.3公里，完成街巷硬化5万平方米，启动达拉特湿地公园建设，推动解决城市内涝问题。巩固国家园林县城创建成果，建成园林苗圃育苗基地，新建城市绿道3.2公里，新增绿地5万平方米，提升绿化景观30万平方米。新建垃圾转运站2座、建筑垃圾场1个，新建改造水冲式公厕10座以上，实施老旧小区节能改造15万平方米，提高城区供热质量。着力推进城市棚改工作。坚持以棚改优化城市功能、提升城市形象，通过政府主导、社会参与等运作模式高质量推进棚户区改造，年内完成2312户房屋征收；积极争取棚改债券资金，推进新的片区棚改项目；鼓励社会力量参与旧城改造。启动建设化肥厂、兴盛园棚改安置小区，争取人和二期公寓楼、馨和家园棚改安置小区建成投用。

开展城市管理提质行动。深化城市执法体制改革，抓好市场秩序、交通秩序、环境卫生等综合整治，加快城区二手车和机动车维修市场“划行归市”，提高城市精细化管理水平。加强街道社区阵地建设，全面推行“网格化”治理，打造“四型”街道和“三好”社区。深入推进物业管理体制改革，加快物业管理规范化、服务标准化。启动全国县级文明城市提名城市创建，深入开展文明小区、健康小区、安全小区建设，抓好市民文明素质养成，培育城市人文精神，努力让城市居民生活更方便、更舒心、更美好。

开展基础设施优化行动。实施黄河二期防洪新增工程，建设光伏基地供水项目。推行农村公路路长制，巩固“四好农村路”全国示范县创建成果，加快推进农村公路、矿区公路和旅游专线建设。加快电网配套，完成城网改造，新建开发区220千伏和110千伏变电站各1座，实施35千伏输变电工程3处，完成2座35千伏变电站增容改造，新增供电线路56.8公里。完善信息网络设施，实施宽带下乡村、电信普遍服务试点和广播影视固边工程。

（六）坚定不移深化改革创新和扩大开放，在激发高质量发展动力活力上迈出新步伐

更大力度推进改革攻坚。深化经济体制、生态文明等领域改革，持续抓好习近平总书记嘱咐内蒙古先行先试的改革。深入推进供给侧结构性改革，巩固“三去一降一补”成果。推动财税、金融、国资国企等重大改革，深化农村土地制度改革，加快农业水价和集体林业综合改革。推进农村集体产权制度改革，完成集体资产清产核资和集体经济组织成员身份确认。围绕提高群众满意度，统筹推进教育、文化等民生领域改革和自选类改革。

更深层次强化创新驱动。完善促进科技创新的政策措施，对企业技术创新给予实质性扶持，加快新奥石墨烯研发中心建设，争取新奥煤基低碳能源国家重点实验室落地。突出企业科技创新主体地位，着力在清洁能源、现代煤化工、新材料等产业上推动关键共性技术攻关，年内新增国家高新技术企业2家，转化科技成果10项以上。加强创新主体培育，新增自治区级以上创新创业平台2家。深化人才强旗战略，优化“1+6+N”人才政策体系，开展“人才引领促发展”行动，培养一批行业领军人才、企业家人才和高技能人才队伍。

更广范围开展合作共建。积极参与“一带一路”和中蒙俄经济走廊建设，扩大中轩黄原胶、亿利PVC、韩廷宴泡菜等特色产品出口规模。加强与京津冀、长三角、珠三角等地合作，提升开放型经济发展水平。继续深化与北京西城区的协作，在农畜产品进京、旅游宣传营销等方面取得新成果。抓住自治区建设沿黄生态经济带、推动呼包鄂榆城市群发展等战略契机，加强与包头等毗邻地区的联动发展，力争在能源开发、基础设施建设等领域取得实质性进展。

（七）坚定不移保障和改善民生，在提高人民群众获得感、幸福感、安全感上迈出新步伐

推动公共服务均等化。落实立德树人根本任务，不断提高教育教学质量，发展公平优质教育；加强师资培训教育，新招聘教师270名，全面优化校长、教师、教研员队伍；统筹推进学校标准化建设，新建续建中小学和幼儿园项目7个，确保蒙古族学校综合楼等项目建成投用；加强校园文明建设，营造健康向上的育人氛围。推进健康达拉特建设，突出预防为主，深入开展爱国卫生运动，倡导健康生活方式；深化医药卫生体制改革，推动分级诊疗和医联体建设，提高医疗卫生服务能力；支持中蒙医药事业发展，启动中蒙医医院二期工程；发展“互联网+医疗健康”，扩大远程医疗服务范围，让群众享受优质医疗服务。鼓励开展徒步、骑行等全民健身运动，提高全民健身水平，新建社区全民健身馆2个、全民健身示范点1处。完善公共文化服务体系，完成博物馆图书馆装修布展，深入开展文化下乡、文化进社区等活动，丰富群众精神文化生活；大力弘扬乌兰牧骑精神，深入农村牧区、街道社区等地送演出、送辅导100场以上，更好地服务基层群众；加大文化遗产保护力度，加强秦汉长城等遗址保护。开展以“六进”为抓手的民族团结创建活动，建成自治区民族团结进步示范旗。

织密织牢社会保障网。把稳就业摆在突出位置，统筹抓好高校毕业生、退役军人、农牧民工等群体就业，年内新增城镇就业4200人，城镇登记失业率控制在3.1%以内。落实全民参保计划，提高养老保险待遇水平。强化兜底保障，城乡低保标准每人每月提高64元和49元，农村特困人员基本生活标准每人每月提高170元。启动殡仪服务中心建设，确保综合福利中心投入使用。常态化走访关爱高龄老人、“空巢”老人，让老年人感受到党和政府的温暖。认真落实上级政策措施，做好退役军人服务保障工作。严格执行“两金五制”制度，依法保障农牧民工合法权益。

营造安全稳定社会环境。完善矛盾纠纷多元化解机制，妥善解决群众反映的突出问题。严守安全生产红线，推进煤矿、化工、消防、道路交通、防凌防汛等重点领域安全检查常态化，全面加强危化品安全综合治理，坚决杜绝重特大安全事故发生。强化食品药品监管，确保群众饮食用药安全。健全餐厨垃圾处理机制，加大动物疫病防控力度，有效防控非洲猪瘟疫情。完善社会稳定风险评估机制，加强突发事件应急管理。扎实开展第四次全国经济普查，做实数据、摸清家底。加强国防后备力量建设，深入开展国防教育活动。推进公共法律服务体系建设，做好“七五”普法、社区矫正等工作，提高社会法治意识。强化社会治安综合治理，建设公安派出所7个，深入推进扫黑除恶专项斗争，严厉打击各类违法犯罪活动，确保社会大局和谐稳定。

三、建设人民满意的服务型政府

（一）坚定方向，忠诚履职。坚持把政治建设摆在首位，树牢“四个意识”，坚定“四个自信”，坚决做到“两个维护”，确保思想上对标对表、行动上紧跟紧随、执行上坚定坚决。严守政治纪律和政治规矩，推动中央、自治区、市重大决策部署在达拉特落地见效。完成政府机构改革，确保职能优化、协同高效。加强政府系统党的建设工作，增强“八个本领”，创新工作思路，务实推动发展。

（二）厉行法治，依法行政。遵守宪法和法律，始终让政府权力在法治轨道上运行。认真落实公众参与、专家论证、风险评估、合法性审查和集体决策制度，提高政府决策的科学性和民主性。坚持“三重一大”事项向旗委常委会报告制度，自觉接受人大法律监督和政协民主监督，切实做好人大代表建议、政协委员提案和检察建议办理工作。严格执行重大决策跟踪评价、终身责任追究和责任倒查机制，用制度管权、管事、管人。深化“三务”公开，及时回应社会关切，提高政府工作透明度和公信力。

（三）担当作为，务实高效。树立担当精神，勇于攻坚克难，实行重点工作和重大项目挂图作战，以“钉钉子”精神推动各项工作落<。强化效能建设，坚持说了就算、定了就干、马上就办，绝不允许在抓落实上“挂空挡”“掉链子”。加强诚信建设，政府要带头讲诚信，做到言出必行、有诺必践。落实容错纠错机制，为勇于负责者负责、为敢于担当者担当。严格督查问效，坚决克服懒政、庸政、怠政。强化96118民生服务热线，着力解决群众反映强烈的热点难点问题。

（四）遵规守纪，廉洁从政。严格落实政府系统从严治党、从严治政主体责任和“一岗双责”，全面履行意识形态工作责任制。巩固拓展作风建设成果，坚定不移纠“四风”、树新风。加大民生资金、政府投资项目审计监督力度，保障资金和项目安全。全力支持监察委员会工作，建立健全监督体系，强化廉政风险防控，始终保持惩治腐败高压态势，坚决整治群众身边腐败问题，努力做到干部清正、政府清廉、政治清明。

各位代表，担当书写辉煌，实干赢得未来。让我们更加紧密地团结在以习近平同志为核心的党中央周围，在旗委的坚强领导下，不忘初心、牢记使命，守正笃实、久久为功，奋力开创新时代达拉特高质量发展新局面，以优异成绩庆祝新中国成立70周年！
